# Acrylamide inhibits long-term potentiation and learning involving microglia and pro-inflammatory signaling

**DOI:** 10.1038/s41598-022-16762-7

**Published:** 2022-07-20

**Authors:** Yukitoshi Izumi, Chika Fujii, Kazuko A. O’Dell, Charles F. Zorumski

**Affiliations:** 1 Departments of Psychiatry, Washington University School of Medicine, St. Louis, MO USA; 2 The Taylor Family Institute for Innovative Psychiatric Research, Washington University School of Medicine, St. Louis, MO USA

**Keywords:** Neuroscience, Physiology, Natural hazards, Neurology

## Abstract

Acrylamide is a chemical used in various industries and a product following high-temperature cooking of vegetables containing asparagine. Environmental or dietary exposure to acrylamide could impair cognitive function because of its neurotoxicity. Using rat hippocampal slices, we tested whether acrylamide alters induction of long-term potentiation (LTP), a cellular model of learning and memory. We hypothesized that acrylamide impairs cognitive function via activation of pro-inflammatory cytokines because robust upregulation of NLRP3 inflammasome has been reported. Although acrylamide up to 3 mM did not alter basal synaptic transmission, incubation with 10 μM or acute administration of 100 μM acrylamide inhibited induction of LTP. Inhibitors of toll-like receptor 4 (TLR4), and minocycline, an inhibitor of microglial activation, overcame the effects of acrylamide on LTP induction. Furthermore, we observed that acrylamide failed to inhibit LTP after administration of MCC950, an inhibitor of NLRP3, or in the presence of Interleukin-1 receptor antagonist (IL-1Ra). We also found that in vivo acrylamide injection transiently impaired body weight gain and impaired one-trial inhibitory avoidance learning. This learning deficit was overcome by MCC950. These results indicate that cognitive impairment by acrylamide is mediated by mechanisms involving microglia and release of cytokines via NLRP3 activation.

## Introduction

Acrylamide (ACR), or 2-propenamide, is a vinyl monomer with the molecular formula C_3_H_5_NO and is widely used to make polyacrylamide, which is used in treating waste water discharge and in industrial processes. In addition, acrylamide and polyacrylamides are used in production of dyes and organic chemicals, contact lenses, cosmetics and toiletries, permanent-press fabrics, paper and textile production, pulp and paper production, ore processing, sugar refining, and as a chemical grouting agent and soil stabilizer for construction of tunnels, sewers, wells and reservoir^1^.

While occupational and environmental exposures to ACR have long been a concern^2^, ACR is also a by-product in the Maillard reaction via condensation of reducing sugars and amino acids such as asparagine, a major amino acid in potatoes, in the process of cooking at high temperatures^3,4^. Thus, excessive dietary intake of ACR in foods such as French fries and potato chips is also a concern for human health.

Exposure to ACR is harmful because of its neurotoxicity, reproductive toxicity, genotoxicity and carcinogenicity^5^. Peripheral neuropathy has been described in workers chronically exposed to ACR^6^. In addition, damage to the central nervous system (CNS) has also been reported^7,8^. Cognitive decline is also suggested in elderly people who ingest greater amounts of ACR over a number of years^9^, suggesting that dietary exposure may cause CNS problems. Indeed, 16 week exposure to ACR in drinking water worsens cognitive deficits in mice^10^ and more chronic exposure (12 months) in rats results in neuronal deficits in the hippocampus and cortex accompanied with poor performance in spatial memory tests^11^.

Although mechanisms underlying ACR neurotoxicity are not fully understood, several hypotheses have been proposed, including inhibition of kinesin-based fast axonal transport, alteration of neurotransmitter levels, and direct inhibition of neurotransmission^12^. More recently, the possible involvement of inflammatory reactions in ACR toxicity is postulated. In animal models, increased levels of circulating lipopolysaccharide (LPS), interleukin-1ß (IL-1β) and tumor necrosis factor-ɑ (TNF-α) after dietary exposure^10^ or robust upregulation of protein levels of nucleotide-binding oligomerization domain-3 (NLRP3) inflammasome constituents including NLRP3, caspase-1 and IL-1β after dietary exposure^11^ were observed. Furthermore, Sui et al. found that 24 h administration of 2.5 mM ACR increases levels of NLRP3 inflammasome constituents in BV2 microglia, suggesting that effects of ACR on NLRP3 can be acute^13^. Interestingly, these changes were inhibited by MCC950, an inhibitor of NLRP3 activation and inflammasome formation^14^.

Taken together, it appears that ACR acutely impairs cognitive functions through direct activation of NLRP3 in the CNS. Using rat hippocampal slices, we examined whether brief exposure to ACR impairs long-term potentiation (LTP), a cellular mechanism of learning and memory, and the possible involvement of pro-inflammatory activation in its acute toxicity.

## Methods

### Animals

Sprague–Dawley albino rats were obtained from Harlan Laboratories (Indianapolis IN) and were housed in approved facilities at Washington University. Animal use followed National Institute of Health (NIH) guidelines and was approved by the Washington University Institutional Animal Care and Use Committee (IACUC). The reporting in the manuscript follows the recommendations in the ARRIVE guidelines.

### Hippocampal slice preparation and physiology

Hippocampal slices were prepared from postnatal day (P) 28–32 male albino rats using previously described methods^15^. Dissected hippocampi were pinned on an agar base in ice-cold artificial cerebrospinal fluid (ACSF) containing (in mM): 124 NaCl, 5 KCl, 2 MgSO_4_, 2 CaCl_2_, 1.25 NaH2PO_4_, 22 NaHCO3, 10 glucose, bubbled with 95% O2–5% CO_2_ at 4–6 °C. The dorsal two-thirds of the hippocampus was cut into 500 µm slices using a rotary slicer. Acutely prepared slices were kept in an incubation chamber containing gassed ACSF for at least 1 h at 30 °C before experiments.

For electrophysiological studies, slices were transferred to a submersion-recording chamber at 30 °C with ACSF and perfused continuously at 2 ml/min. Extracellular recordings were obtained from the apical dendritic layer (*stratum radiatum*) of area CA1 for monitoring excitatory postsynaptic potentials (EPSPs) with electrodes filled with 2 M NaCl (5–10 MΩ resistance).

Because LTP is a synaptic phenomenon, we focused on recordings of EPSP slope. EPSPs were evoked using 0.1 ms constant current pulses through a bipolar stimulating electrode in the Schaffer collateral (SC) pathway. Responses were monitored by applying single stimuli every 60 s at half-maximal intensity based on a control input–output (IO) curve. In some slices, we also examined the paired pulse ratio evoked by two stimuli at 21 ms interval to detect changes in probability of presynaptic vesicular release. After obtaining stable baseline recordings for at least 10 min, LTP was induced by a single 100 Hz × 1 s high frequency stimulation (HFS) using the same intensity stimulus. Following HFS, responses were monitored by single stimuli once per minute during the period of post-tetanic potentiation (PTP) and then every five minutes for the remainder of an experiment. For display purposes, graphs show data every 5 min except during initial post-tetanic potentiation. In some experiments, long-term depression (LTD) was induced by 1 Hz × 900 pulse low frequency stimulation (LFS)^16^.

Isolated EPSPs mediated by N-methyl-D-aspartate receptors (NMDAR)s were recorded at very low frequency SC stimulation (1/min) in ACSF containing 0.1 mM Mg^2+^ and 2.5 mM Ca^2+^ to promote NMDAR activation and 30 µM 6-cyano-7-nitroquinoxaline-2,3-dione (CNQX), to eliminate the contribution of AMPARs^16^. This NMDA-EPSP is completely blocked by 2-amino-5-phosphovareic acid (APV), a selective NMDAR antagonist^17^.

### Injection of acrylamide and behavioral studies

Rats were tested for memory acquisition in a one-trial inhibitory avoidance learning task^18–20^. This task reflects explicit-declarative fear memories and has been associated with hippocampal LTP; the task is relatively simple to administer with high reliability and clear behavioral endpoints^21–23^. The testing apparatus consists of two chambers, only one of which is lit. Both compartments have a floor of stainless steel rods (4 mm diameter, spaced 10 mm apart) through which an electrical shock could be delivered in the dark chamber (12 × 20 × 16 cm). The adjoining lit compartment (30 × 20 × 16 cm) was illuminated with four 13 W lights. Light intensity in the lit chamber was 1000 lx while that in the dark chamber was < 10 lx. On the first day of testing, rats were placed in the lit chamber and allowed to habituate to the apparatus by freely moving between chambers for 10 min without any foot shocks being administered. On the next day, mice were administered ACR (100 mg/kg ip) or vehicle (saline) 1 h prior to training. MCC950 (50 mg/kg ip) was injected 24 h and 2 h before ACR administration. It has been reported that one time injection of a similar dose of acrylamide to rats induces neurological sequelae^24^. At the time of training, animals were initially placed in the lit compartment and allowed to explore the apparatus freely for up to 300 s (5 min). When rats completely entered the dark chamber, they were immediately given a foot shock. Upon returning to the lit chamber, rats were removed from the apparatus and returned to their home cages. On the next day of testing, rats were placed in the lit chamber without any drug treatment and the latency to enter the dark compartment was recorded over a 300 s trial.

### Chemicals

TAK-242 (CAS 243984-11-4 Cat 6587/5) was from R&D Systems (Minneapolis MN). LPS-RS (from *Rhodobacter sphaeroides,* Catalog # tlrl-rslps) and MCC950 (CAS 210826-40-7, Catalog # inh-mcc) were purchased from InvivoGen (San Diego CA). Other chemicals, including minocycline (CAS 13614-98-7, Cat# M2280000) and IL1-Ra (Cat# SRP 3084), and salts were obtained from Millipore Sigma Chemical Company. Drugs were prepared as stock solutions in either ACSF or DMSO and diluted to final concentration at the time of experiment. The concentrations of TAK-242, LPS-RS and minocycline are based on our previous studies using those inhibitors against LPS^23^. The dose and concentrations of MCC950 and IL1-Ra followed studies by Coll et al.^14^ and Edy et al.^25^, respectively.

### Statistical analysis

Physiological data were collected and analyzed using PClamp software (Molecular Devices, San Jose CA). Data are expressed as mean ± SEM 60 min following HFS, and are normalized with respect to initial baseline recordings (taken as 100%). Statistical comparisons in physiological studies were based on IO curves at baseline and sixty minutes following HFS to determine the degree of change in EPSP slope at the 50% maximal point with p < 0.05 considered significant. Data in figures for physiological studies are from continuous monitoring of EPSPs at low frequency during the course of experiments and thus may differ from numerical results described in the text, which represent analyses based on comparison of input–output curves. Statistics were performed using commercial software (GraphPad Prism 9.2.0, GraphPad Software, La Jolla California). For comparisons of LTP results with 100 µM ACR alone, or one-trial learning after injection of 100 mg/kg ACR, data were analyzed by two-way analysis of variance (ANOVA) followed by Dunnett’s multiple comparison test. In other studies, a two-tailed Student’s t-test was used for comparisons between groups. In cases of non-normally distributed data, the non-parametric Wilcoxon Rank Sum Test was used.

## Results

### ACR inhibits hippocampal LTP

**Figure 1:**
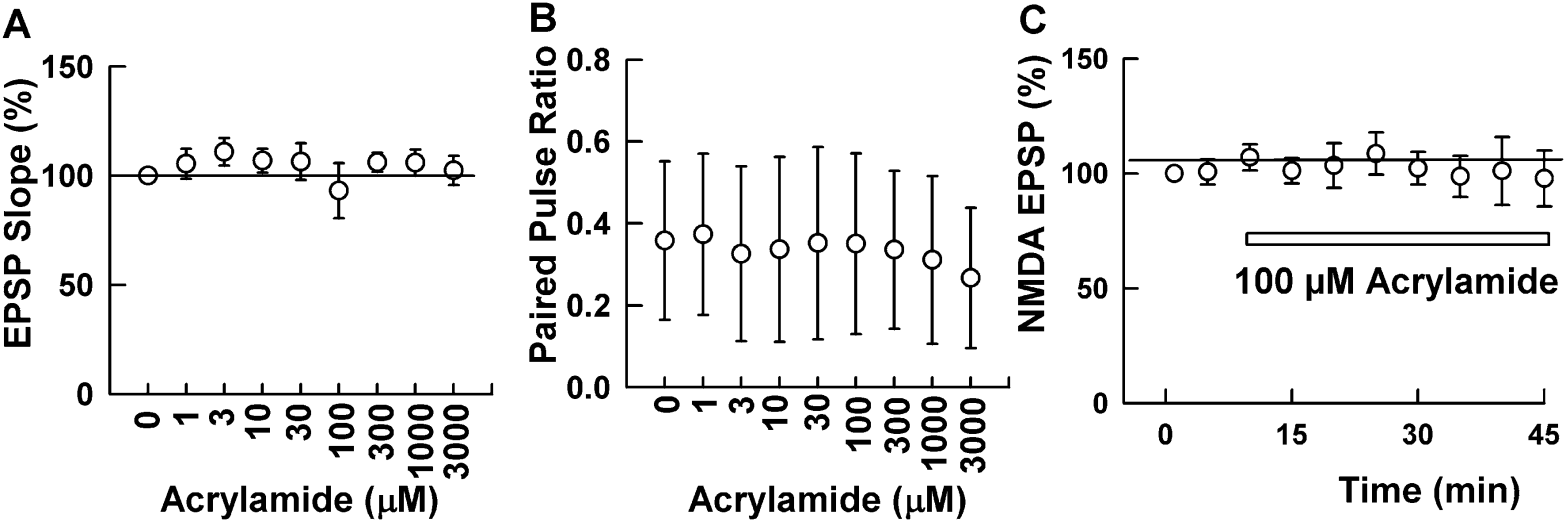
ACR does not alter basal synaptic transmission in the CA1 region of hippocampal slices. (**A**) In 5 slices, the concentration of ACR was raised step by step every 15 min. EPSPs, evoked by stimulation of the Schaffer collateral pathway, were not altered by ACR up to 3 mM. (**B**) Similarly, paired pulse depression normally observed at 21 ms was not altered by ACR up to 3 mM. (**C**) In different slices, NMDAR-mediated synaptic responses recorded in low magnesium were not altered by 100 μM ACR.

In initial studies, we examined acute exposure to ACR in ex vivo hippocampal slices to determine effects on synaptic function and long-term plasticity in the CA1 region. When ACR was perfused in increasing concentrations every 15 min, we observed no significant changes in baseline transmission at concentrations up to 3 mM (N = 5, Fig. 1A). Using paired stimulation at an interval of 21 ms, we also analyzed whether ACR altered short-term plasticity. ACR did not significantly alter the ratio of the 2nd EPSP slope to the 1st EPSP slope for either dendritic or somatic EPSPs at any concentration tested (p = 0.124, one-way ANOVA, N = 5 per group, Fig. 1B). These studies indicate that ACR does not significantly affect basal synaptic function at concentrations up to 3 mM. We also examined the effects of ACR on NMDA receptor (NMDAR)-mediated synaptic responses because NMDAR activation is pivotal for induction of long-term forms of synaptic plasticity in the CA1 region and inhibition of NMDA receptors would result in impairment of synaptic plasticity. Administration of 100 μM ACR, however, did not acutely alter NMDAR-mediated EPSPs (105.2 ± 9.3% after 30 min administration, N = 5 Fig. 1C).

**Figure 2:**
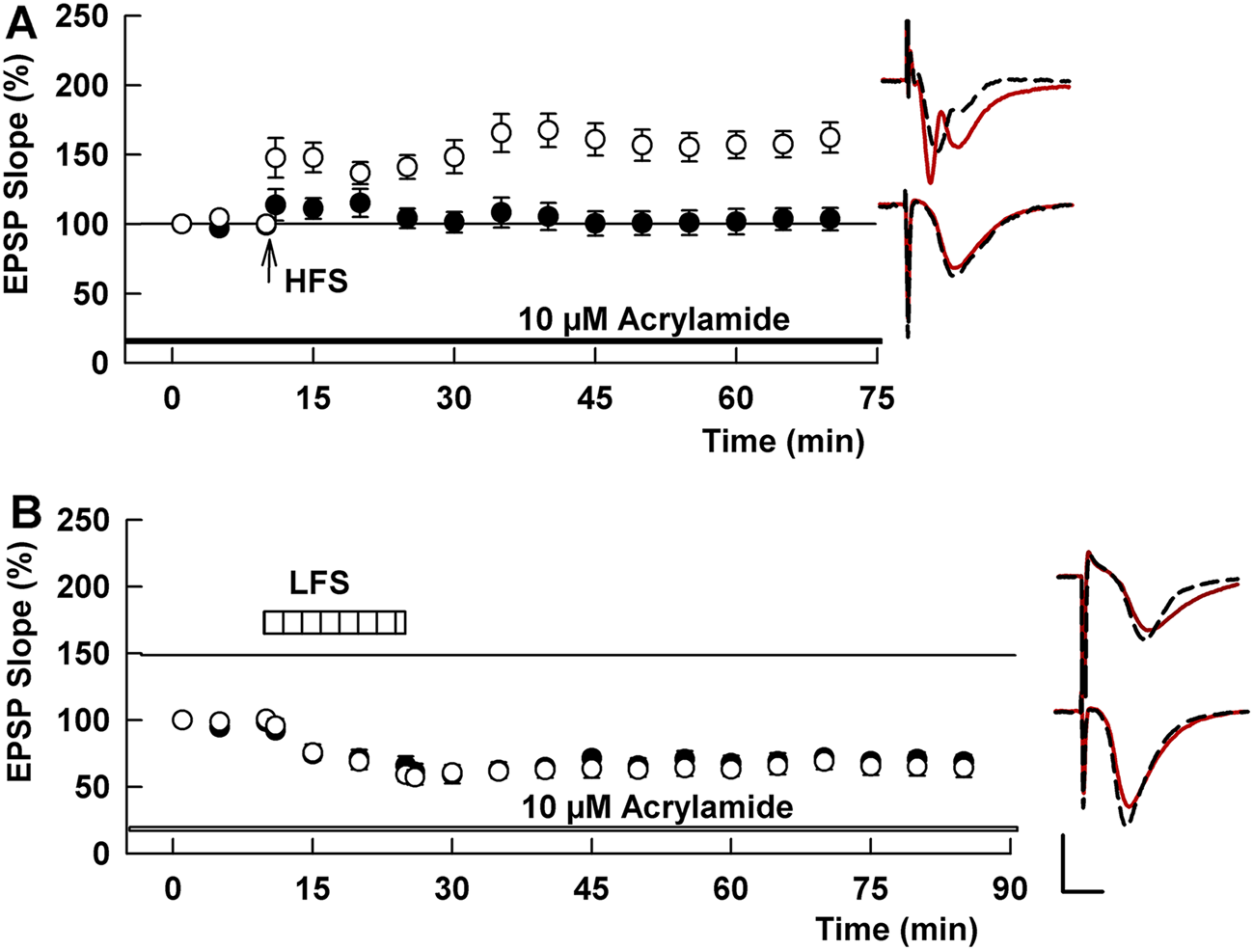
ACR inhibits LTP, but not LTD, in the CA1 region of rat hippocampal slices. (**A**) Ten μM ACR when administered for 4 h or more prior to and during slice recording (black bar) blocks LTP induction (black circles) after a single 100 Hz × 1 s HFS (arrow). Control LTP in naive hippocampal slices is shown in white circles. Traces to the right of this graph and in subsequent figures show representative EPSPs at baseline before HFS (dashed traces) and 60 min following HFS (solid red lines). Calibration bar (Panel B): 1 mV, 5 ms. (**B**) Ten μM ACR (black bar) does not inhibit LTD induction after LFS (hatched bar). Control LTD is shown in white circles. Traces show representative EPSPs at baseline before LFS (dashed traces) and 60 min following LFS (solid red lines).

We next examined whether ACR alters induction of long-term plasticity. In the absence of ACR, HFS consistently induced LTP in control slices (158.3 ± 4.6% of baseline measured 60 min after HFS, N = 5, Fig. 2A). To examine effects of ACR on synaptic plasticity, we initially incubated slices with 10 μM ACR for 4–5 h. Since it has been shown that some fried meals could contain over 10 μmol/kg ACR^26^, we started from this concentration. Ten μM ACR was administered prior to delivering a 100 Hz × 1 s HFS (for LTP) or 1 Hz × 900 pulse LFS (for LTD); these stimuli reliably induce long-term plasticity in control slices from P30 rats. Using this subacute ACR exposure, LTP was not induced by HFS (96.5 ± 5.2% of baseline, N = 5), although LTD was successfully induced by LFS (62.5 ± 5.2%, N = 4, Fig. 2A,B).

**Figure 3:**
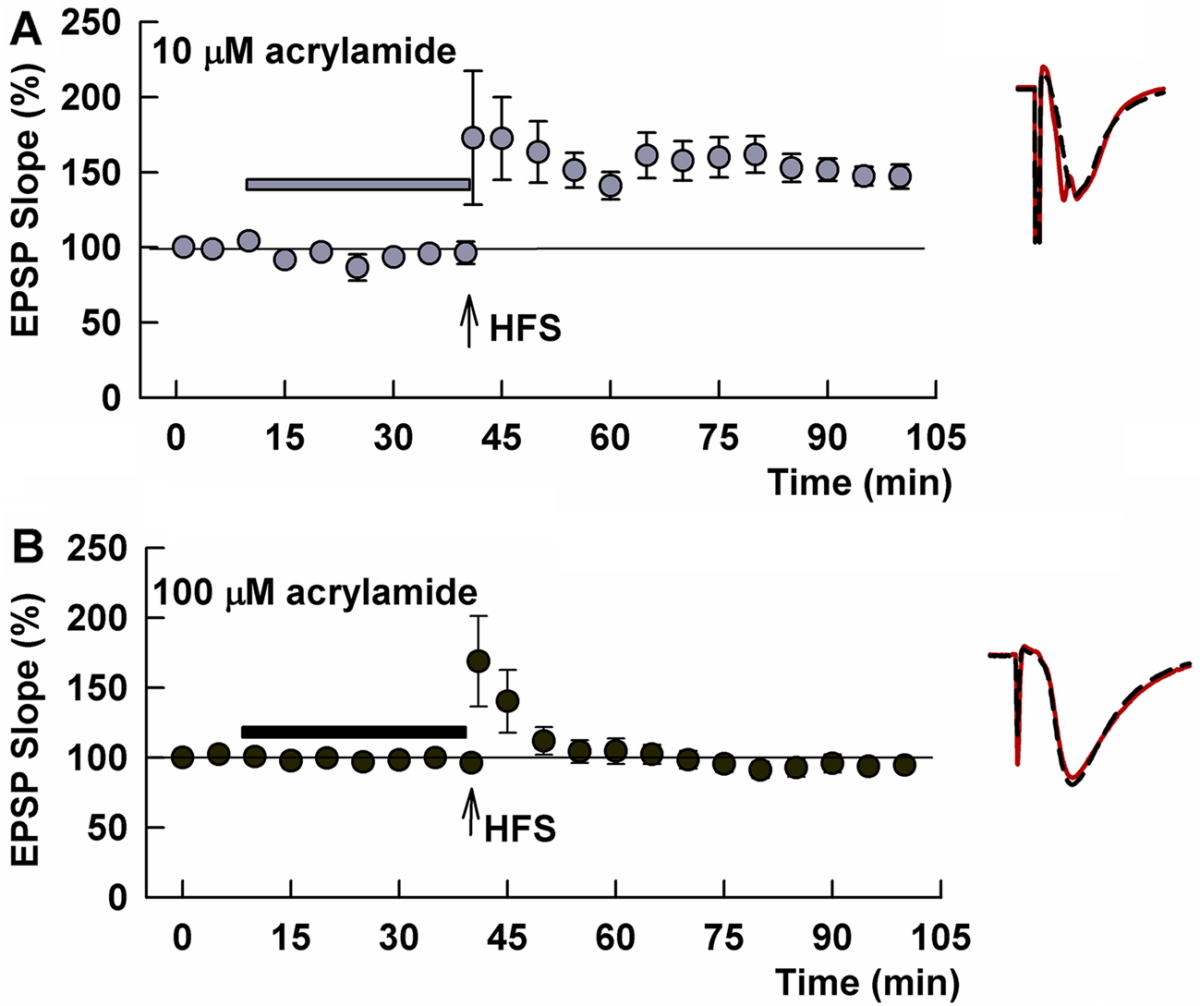
ACR acutely inhibits LTP in the CA1 region of rat hippocampal slices. (**A**) When administered for 30 min, 10 µM ACR (gray bar) does not inhibit the induction of LTP in response to HFS (arrow) (gray circles). (**B**) 100 µM ACR (black bar) acutely inhibits LTP (black bar). Traces to the right of these graphs show representative EPSPs at baseline before HFS (dashed traces) and 60 min following HFS (solid red lines). Calibration bar (Panel B): 1 mV, 5 ms.

We also examined whether a shorter exposure to ACR alters LTP induction. Although 30 min administration of 10 μM ACR did not inhibit LTP (133.1 ± 18.0%, N = 5, Fig. 3A), 100 μM ACR acutely inhibited LTP induction (97.7 ± 5.8%, N = 10, P = 0.0014 vs control, Fig. 3B). In another set of slices, LFS delivered following 30 min administration of 100 µM ACR successfully induced LTD (52.7 ± 6.1%, N = 5, not shown), indicating again that LTD is not affected by exposure to ACR.

### ACR inhibits LTP via pro-inflammatory pathways

**Figure 4:**
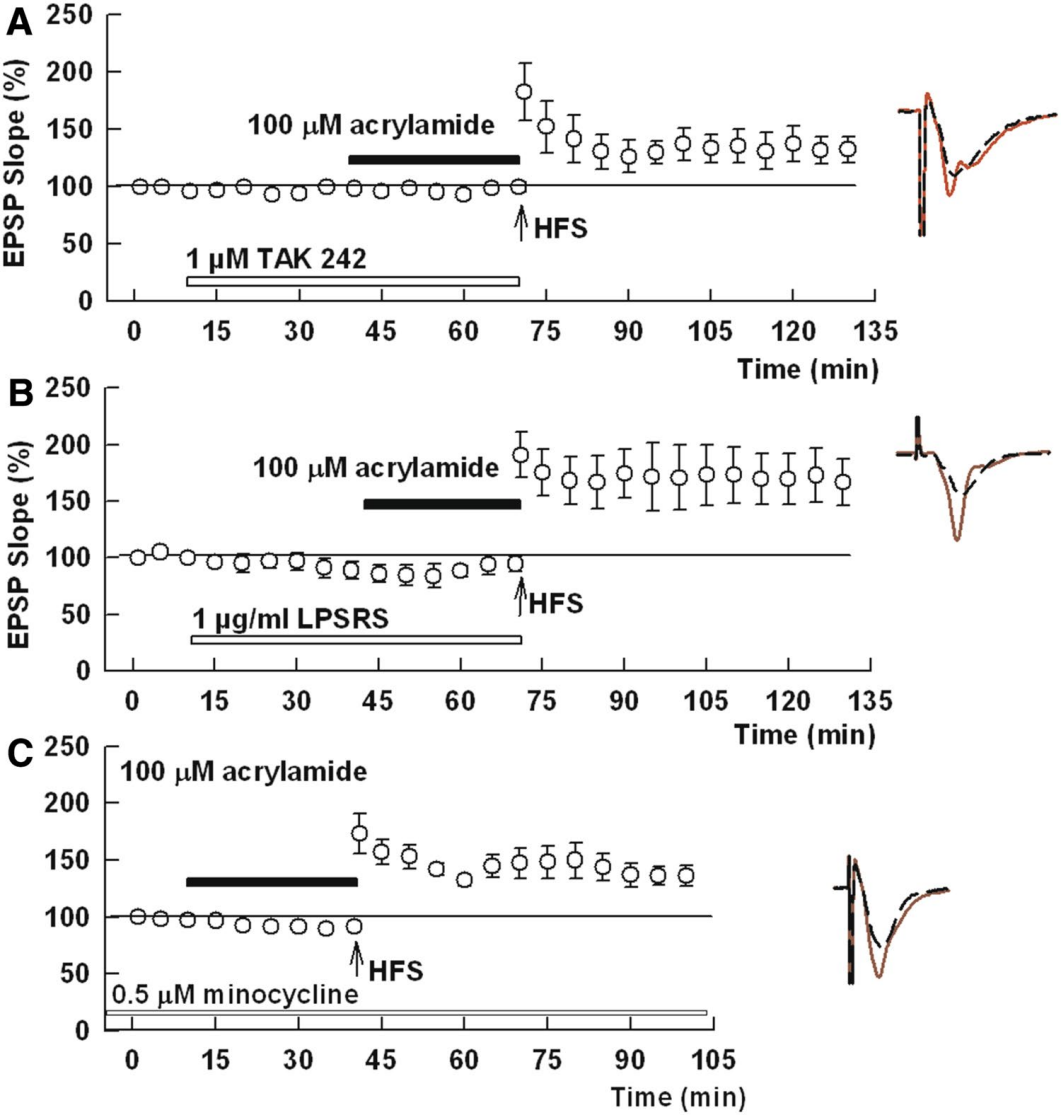
TLR4 antagonists and minocycline overcome effects of ACR on LTP. (**A**) TAK 242 (white bar), a TLR4 antagonist, allows LTP induction after HFS (arrow) in spite of the presence of 100 µM ACR (black bar). (**B**) Similarly, LPSRS (white bar), another TLR4 antagonist, overcomes inhibitory effects of 100 µM ACR on LTP induction. (**C**) At 0.5 µM, the microglial inhibitor, minocycline (white bar) also overcomes the inhibitory effects of ACR (black bar) on LTP. Traces show representative EPSPs. Calibration: 1 mV, 5 ms.

Based on prior studies indicating that ACR can evoke inflammatory responses^11,13^, we examined the role of pro-inflammatory pathways in the effects of ACR. To test the involvement of pro-inflammatory signaling, we used TAK-242, a specific inhibitor of toll-like receptor 4 (TLR4), and LPSRS, an agent that also inhibits TLR4 in addition to non-canonical inflammasome activation^23^. In the presence of 1 μM TAK-242, administration of 100 μM ACR did not inhibit LTP induction (156.9 ± 12.7%, N = 9, P = 0.0014 vs. ACR alone, Fig. 4A). Similarly, 3 μg/ml LPSRS, overcame the inhibitory effects of ACR (160.9 ± 11.5%, N = 5, P = 0.0007 vs. ACR alone, Fig. 4B). We then examined effects of minocycline, an inhibitor of microglial activation^23^. In the presence of 0.5 µM minocycline, 100 µM acrylamide failed to inhibit LTP induction (138.7 ± 4.2, N = 6, P = 0.0277, Fig. 4C). These findings strongly suggest that TLR4 activation, likely in microglia, is involved in ACR-mediated inhibition of LTP induction.

**Figure 5:**
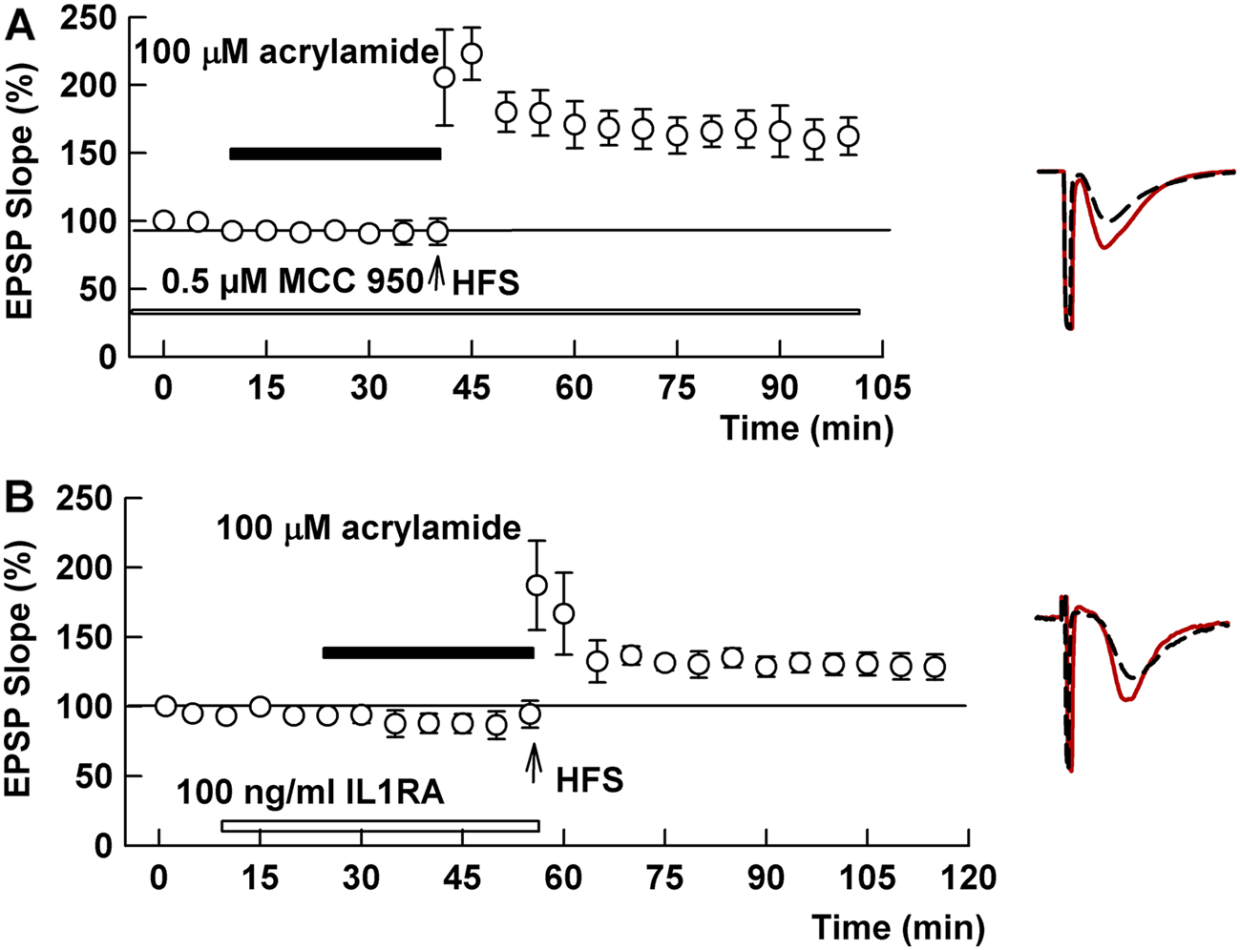
An NLRP3 inhibitor and IL-1 receptor antagonist overcome effects of ACR on LTP. (**A**) Prolonged administration of 0.5 μM MCC950, an inhibitor of NLRP3 for 2–4 h (white bar) prior to HFS (arrow) allows LTP induction in spite of the presence of 100 µM ACR (black bar). (**B**) Similarly, in the presence of 100 ng/ml Interleukin-1 receptor antagonist (white bar), ACR fails to block LTP induction. Traces show representative EPSPs. Calibration: 1 mV, 5 ms.

One of the major consequences of TLR4 activation is stimulation of the NLRP3 inflammasome and release of IL-1^27–30^. Supporting this possibility, we observed that 100 μM ACR failed to inhibit LTP induction in the presence of 0.5 μM MCC950, an inhibitor of NLRP3 (141.8 ± 17.5, N = 5, P = 0.0247 vs. ACR alone, Fig. 5A). Similarly, 100 μM ACR did not block LTP in the presence of 100 ng/ml Interleukin-1 receptor antagonist (IL-1Ra) (132.4 ± 9.3%, N = 8, P = 0.0492 vs. ACR alone, Fig. 5B).

### ACR dampens learning via NLRP3 activation

**Figure 6:**
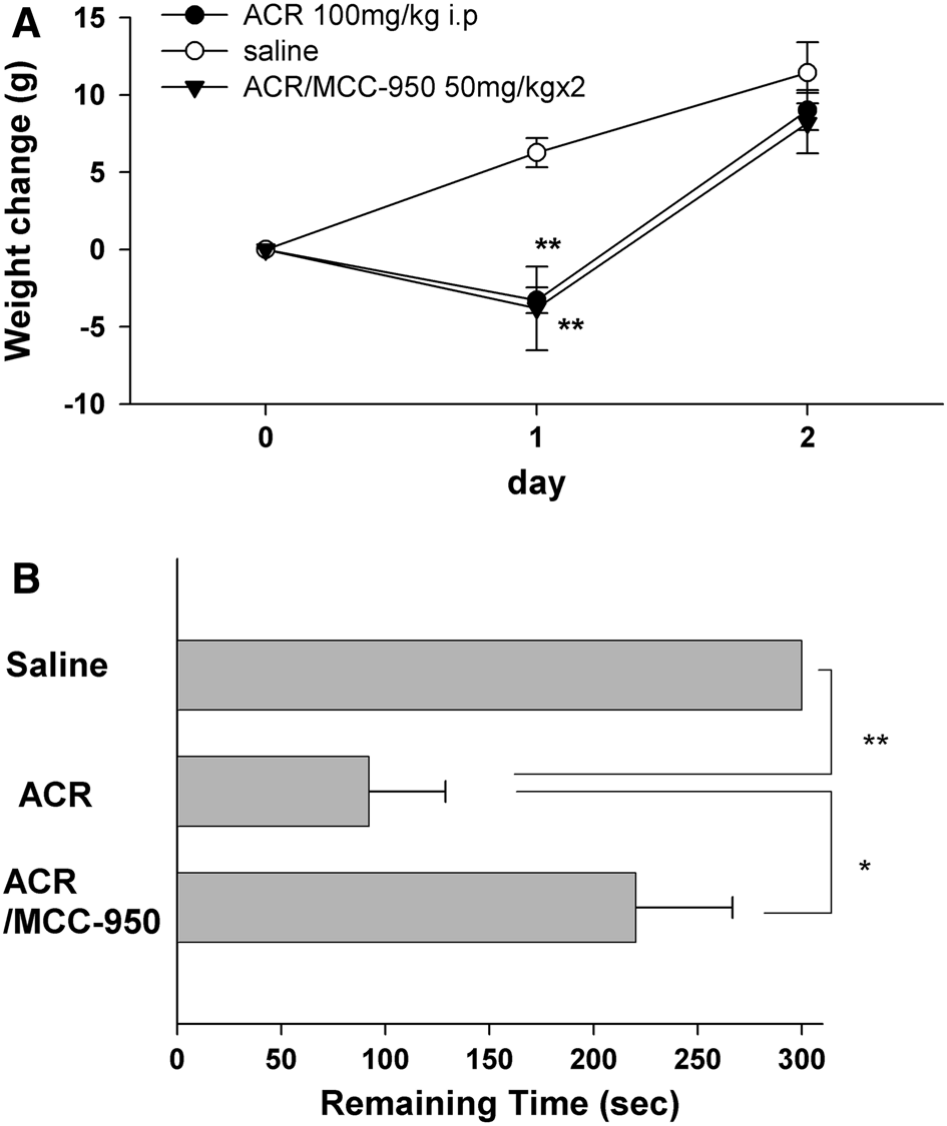
Effects of ACR ip injection in vivo. (**A**) In vivo administration of ACR (100 mg/kg ip) to young rats results in weight loss next day, which is compensated the following day. Injection of MCC950 (50 mg/kg, 24 h and 2 h before ACR administration) did not alter the change in weight gain. **p < 0.01 compared with control by Student *t*-test. (**B**) ACR administered one day prior to inhibitory avoidance training results in a defect in learning as manifest by rats more readily leaving the lit chamber to enter the dark chamber where they had received a foot shock one day previously. The learning defect was overcome by administration of MCC950. **p < 0.01.

In subsequent studies, we examined the effects of systemic ACR on learning and memory using a one-trial inhibitory avoidance task that has been linked previously to hippocampal LTP^20^. ACR was injected (100 mg/kg, i.p.) 24 h before conditioning. The increase in body weight typically observed in juvenile (~ P30) rats was dampened by ACR one day after injection (P = 0.0002) though it returned to control levels on the second day following ACR treatment (P = 0.8832, Fig. 6A). Rats exhibited no noticeable changes in gait or coordination following ACR. ACR treatment had marked acute effects on performance in one-trial learning compared to saline-treated controls when tested 24 h after conditioning. The ACR-induced defect in learning was manifest by rats more readily entering the dark chamber where they had been shocked, whereas controls remained in the lit compartment for the full duration of the trial P = 0.005, N = 8, Fig. 6B).

In rats treated with MCC950 (50 mg/kg i.p. twice), similar dampening of body weight one day after injection was observed but recovered to control levels on the second day (Fig. 6A). In spite of the effect on weight gain, ACR did not impair one-trial learning after injection of MCC950 (P = 0.0454 vs. ACR alone, N = 8, Fig. 6B).

## Discussion

A major finding in this study is the alteration of cognitive function 24 h after in vivo systemic administration of ACR. Effects of ACR on learning and memory are not the result of gross changes in neurological function because we also observed that systemic ACR had no clear effect on gait, activity or level of consciousness. We did observe that ACR temporarily decreased weight gain by 24 h after administration, although ACR-treated rats caught up with controls by 48 h after exposure. A decrease in weight gain was also observed previously in mice treated with ACR^13^.

Cognitive impairment by ACR may be induced indirectly through chronic damage to the blood brain barrier (BBB), and ACR slowly impairs transport and secretary functions of the BBB^31^. However, acute actions following systemic injection suggest that some effects of ACR may be more direct in the CNS and may be detected shortly after administration in ex vivo systems. Indeed, in hippocampal slices treated with 10 μM ACR for several hours, LTP, a cellular model of learning and memory, was markedly impaired. If ACR is distributed equally in the CNS after injection, the concentration of 10 μM suggests that single exposure at 100 mg/kg could have deleterious effects on cognitive function. Although inhibitory effects on LTP induction are also observed when 100 μM ACR was administered for 30 min prior to the delivery of HFS, concentrations of ACR as high as 3 mM did not affect basal synaptic responses or short-term paired-pulse plasticity. These observations suggest that neurotoxic effects of ACR, even after high dose exposure, can be relatively subtle since basic neuronal function is preserved. In contrast, learning dysfunction can occur fairly soon after ACR exposure even if it is only a single exposure. Learning disability is often difficult to detect because of lack of specific symptoms. Even if cumulative levels of ACR are within a low micromolar range, however, learning difficulties may follow repeated consumption of ACR rich foods or environmental exposures.

In the hippocampus, LTP and LTD require activation of NMDA receptors for induction. However, NMDA-EPSPs were not altered by 100 μM ACR, indicating that inhibition of LTP induction by ACR is not through block of NMDA receptors, and, consistent with this conclusion, ACR failed to alter LTD induction. LTP induction can be dampened by other stressors via different mechanisms. For instance, untimely activation of NMDA receptors can inhibit LTP induction^32^. Similarly, toxic cellular stressors such as ethanol and acetaldehyde also inhibit LTP induction^19,33^. Recently, we showed that ISRIB, an inhibitor of cellular integrated stress responses, and GW 3965, an agonist for liver X receptors, do not alter NMDA responses but overcome inhibitory effects of ethanol on CA1 synaptic plasticity^19^. The NLRP3 inflammasome is also involved in stress responses^34^. Because the integrated stress response controls production of mitochondrial reactive oxygen species (mtROS), and NLRP3 inflammasome activation by lipids^35^, it is plausible that cellular stress triggered by neurotoxins activates NLRP3. Indeed, recent studies show potential roles of NLRP3 activation in ACR toxicity. In Kupffer cells, 0.5–1 mM ACR induces ROS production in 24 h and increases expression of NLRP3 and IL-1β mRNA, which results in NLRP3 inflammasome formation^36^. In rats, chronic (12 month) exposure to ACR (5 mg/kg/day) markedly upregulates protein levels of NLRP3 inflammasome constituents including NLRP3 caspase-1, and pro-IL-1β and IL-1β in hippocampus and frontal cortex^11^.

Our results indicate that disruption of LTP by ACR is mediated by TLR4. Expression of TLR4 is not limited to glia but is also observed in neurons, and activation of neuronal TLR4 results in liberation of tumor necrosis factor-α (TNFα) and IL-6^37^. In the present study, however, the action of ACR was clearly prevented by minocycline, which is commonly used to inhibit microglial activation^23^, suggesting that disruption of LTP involves microglia. The disruption of LTP by ACR also involves stimulation of pro-inflammatory signaling via the NLRP3 inflammasome. MCC950 specifically inhibits the NLRP3 inflammasome^38^ and blocks canonical, non-canonical and alternative NLRP3 activation^14,39,40^. Thus, MCC950 is a useful agent to prevent effects of ACR regardless of the pathway by which ACR activates NLRP3^41^. Sui et al. have shown that in BV2 microglia, 2.5 mM ACR for 24 h triggers NLRP3 inflammasome activation and this activation is reversed by 0.1 μM MCC950^13^. They also showed that gait abnormalities observed 7–10 days after ACR exposure were partially attenuated by MCC950, indicating that NLRP3 inflammasome activation is involved in neurological sequelae. Furthermore, MCC950 also dampens NLRP3 activation and cognitive impairment triggered by isoflurane in mice^42^. In the current study, we observed that MCC950 prevents the inhibitory effects of ACR on LTP induction and one-trial avoidance learning. This finding suggests that ACR impairs cognitive functions via NLRP3 inflammasome activation.

NLRP3 inflammasome activation results in secretion of both IL-1 and IL-18. In BV-2 microglia, ACR induces overexpression of both cytokines^13,43^. However, secretion of these cytokines differ in terms of activation pathway and timing^44,45^. It is well known that IL-1 inhibits LTP^46–48^. Thus, it is possible that both cytokines are involved in the inhibitory effects of ACR on LTP induction.

**Figure 7:**
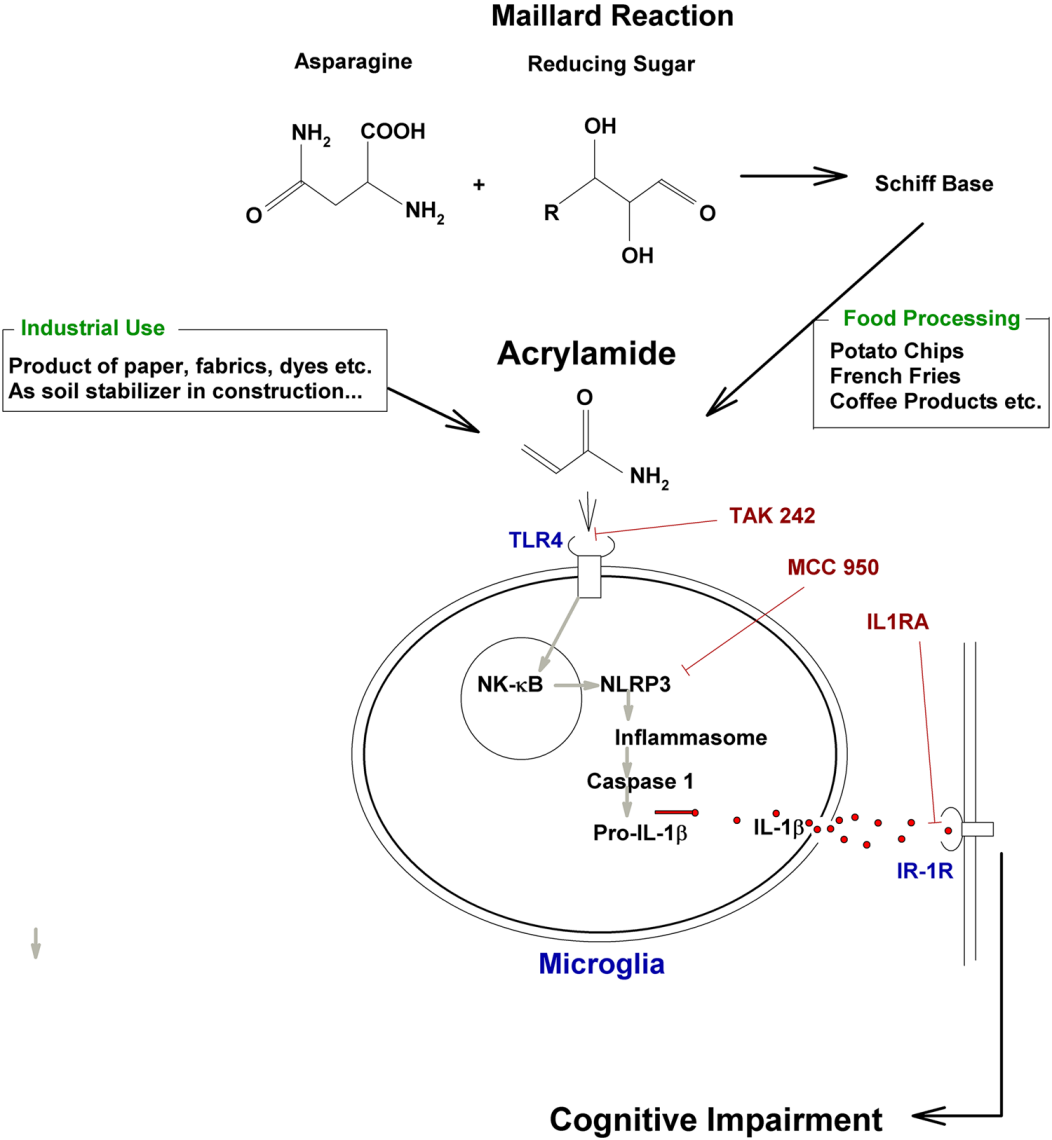
Schematic diagram to show how humans are exposed to ACR and a possible mechanism how ACR impairs cognitive function via activation of TLR4 and the NLRP3 inflammasome in microglia. Brown lines show locations where inhibitors work.

To examine whether IL-1 participates in the action of ACR, we used the interleukin-1 receptor antagonist (IL-1Ra), which selectively binds IL-1 receptors without inducing intracellular responses^49^. Because it has been reported that IL-1Ra alone, given as an intracerebroventricular injection, could inhibit LTP induction in rats^47^, or because IL-1Ra may depotentiate established LTP^50^, IL-Ra was administered before, but not after HFS in the current study. Similar to MCC950, IL-1Ra clearly prevented the inhibitory effects of ACR on LTP induction. This result suggests a critical role for IL-1 in the inhibition of LTP induction following ACR exposure. Further studies will be needed to determine whether there is also a role for IL-18. Possible mechanisms underlying how ACR impairs cognitive function are summarized in Fig. 7.

Taken together, our results indicate that ACR can impair cognition and hippocampal function, in addition to its other known effects as a carcinogen. Learning problems may result from excessive exposure to ACR through activation of proinflammatory signaling (Fig. 7).

### Ethical approval and consent to participate

The animals used in this study were housed in approved facilities at Washington University. Animal use followed National Institute of Health (NIH) guidelines and was approved by the Washington University Institutional Animal Care and Use Committee (IACUC).

## Data Availability

The datasets used and/or analysed during the current study available from the corresponding author on reasonable request.

## Abbreviations

ACR: Acrylamide
ACSF: Artificial cerebrospinal fluid
ANOVA: Analysis of variance
APV: 2-Amino-5-phosphovareic acid
BBB: Blood brain barrier
CNQX: 6-Cyano-7-nitroquinoxaline-2,3-dione
CNS: Central nervous system
EPSP: Excitatory postsynaptic potentials
HFS: High frequency stimulation
IACUC: Institutional Animal Care and Use Committee
IL-1: Interleukin-1
IO: Input–output
i.p.: Intraperitoneally
IR-Ira: Interleukin-1 receptor antagonist
LFS: Low-frequency stimulation
LPS-RS: Lipopolysaccharide from Rhodobacter sphaeroides
LTD: Long-term depression
LTP: Long-term potentiation
mtROS: Mitochondrial reactive oxygen species
NIH: National Institute of Health
NLRP3: NOD-, LRR- and pyrin domain-containing protein 3
NMDAR: N-methyl-D-aspartate receptor
P: Postnatal day
PTP: Post-tetanic potentiation
TNF-α: Tumor necrosis factor-ɑ

## References

[1] PubChem. Acrylamide [Internet]. [cited 2021 Dec 28]. https://pubchem.ncbi.nlm.nih.gov/compound/6579.

[2] Hagmar L, Törnqvist M, Nordander C, Rosén I, Bruze M, Kautiainen A (2001). Health effects of occupational exposure to acrylamide using hemoglobin adducts as biomarkers of internal dose. Scand. J. Work Environ. Health..

[3] Mottram DS, Wedzicha BL, Dodson AT (2002). Acrylamide is formed in the Maillard reaction. Nature.

[4] Stadler RH, Blank I, Varga N, Robert F, Hau J, Guy PA (2002). Acrylamide from Maillard reaction products. Nature.

[5] Exon JH (2006). A review of the toxicology of acrylamide. J. Toxicol. Environ. Health B Crit. Rev..

[6] Pennisi M, Malaguarnera G, Puglisi V, Vinciguerra L, Vacante M, Malaguarnera M (2013). Neurotoxicity of acrylamide in exposed workers. Int. J. Environ. Res. Public Health.

[7] Kuperman AS (1958). Effects of acrylamide on the central nervous system of the cat. J. Pharmacol. Exp. Ther..

[8] Lehning EJ, Balaban CD, Ross JF, LoPachin RM (2003). Acrylamide neuropathy. II. Spatiotemporal characteristics of nerve cell damage in brainstem and spinal cord. Neurotoxicology.

[9] Liu ZM, Tse LA, Chen B, Wu S, Chan D, Kowk T (2017). Dietary acrylamide exposure was associated with mild cognition decline among non-smoking Chinese elderly men. Sci. Rep..

[10] Tan X, Ye J, Liu W, Zhao B, Shi X, Zhang C (2019). Acrylamide aggravates cognitive deficits at night period via the gut-brain axis by reprogramming the brain circadian clock. Arch. Toxicol..

[11] Liu Y, Zhang X, Yan D, Wang Y, Wang N, Liu Y (2020). Chronic acrylamide exposure induced glia cell activation, NLRP3 infl-ammasome upregulation and cognitive impairment. Toxicol. Appl. Pharmacol..

[12] Erkekoglu P, Baydar T (2014). Acrylamide neurotoxicity. Nutr. Neurosci..

[13] Sui X, Yang J, Zhang G, Yuan X, Li W, Long J (2020). NLRP3 inflammasome inhibition attenuates subacute neurotoxicity induced by acrylamide in vitro and in vivo. Toxicology.

[14] Coll RC, Hill JR, Day CJ, Zamoshnikova A, Boucher D, Massey NL (2019). MCC950 directly targets the NLRP3 ATP-hydrolysis motif for inflammasome inhibition. Nat. Chem. Biol..

[15] Tokuda K, Izumi Y, Zorumski CF (2011). Ethanol enhances neurosteroidogenesis in hippocampal pyramidal neurons by paradoxical NMDA receptor activation. J. Neurosci..

[16] Izumi Y, Nagashima K, Murayama K, Zorumski CF (2005). Acute effects of ethanol on hippocampal long-term potentiation and long-term depression are mediated by different mechanisms. Neuroscience.

[17] Izumi Y, Auberson YP, Zorumski CF (2006). Zinc modulates bidirectional hippocampal plasticity by effects on NMDA receptors. J. Neurosci..

[18] Izumi Y, Mennerick SJ, Doherty JJ, Zorumski CF (2021). Oxysterols modulate the acute effects of ethanol on hippocampal N-methyl-d-aspartate receptors, long-term potentiation, and learning. J. Pharmacol. Exp. Ther..

[19] Izumi Y, Zorumski CF (2020). Inhibitors of cellular stress overcome acute effects of ethanol on hippocampal plasticity and learning. Neurobiol. Dis..

[20] Whitlock JR, Heynen AJ, Shuler MG, Bear MF (2006). Learning induces long-term potentiation in the hippocampus. Science.

[21] Parent MB, West M, McGaugh JL (1994). Memory of rats with amygdala lesions induced 30 days after footshock-motivated escape training reflects degree of original training. Behav. Neurosci..

[22] Tokuda K, O’Dell KA, Izumi Y, Zorumski CF (2010). Midazolam inhibits hippocampal long-term potentiation and learning through dual central and peripheral benzodiazepine receptor activation and neurosteroidogenesis. J. Neurosci..

[23] Izumi Y, Cashikar A, Krishnan K, Paul SM, Covey DF, Mennerick SJ, et al. A Pro-inflammatory stimulus disrupts hippocampal plasticity and learning via microglial activation and 25-hydroxycholesterol. J. Neurosci. Off. J. Soc. Neurosci. 2021;JN-RM-1502–21. 10.1523/JNEUROSCI.1502-21.2021 PMC8660051 34725187

[24] Gold BG, Griffin JW, Price DL (1985). Slow axonal transport in acrylamide neuropathy: Different abnormalities produced by single-dose and continuous administration. J. Neurosci..

[25] Edye ME, Brough D, Allan SM (2015). Acid-dependent interleukin-1 (IL-1) cleavage limits available pro-IL-1β for caspase-1 cleavage. J. Biol. Chem..

[26] Powers SJ, Mottram DS, Curtis A, Halford NG (2021). Progress on reducing acrylamide levels in potato crisps in Europe, 2002 to 2019. Food Addit. Contam. Part Chem. Anal. Control Expo. Risk. Assess..

[27] Yang Y, Wang H, Kouadir M, Song H, Shi F (2019). Recent advances in the mechanisms of NLRP3 inflammasome activation and its inhibitors. Cell Death Dis..

[28] Liu Y, Dai Y, Li Q, Chen C, Chen H, Song Y (2020). Beta-amyloid activates NLRP3 inflammasome via TLR4 in mouse microglia. Neurosci. Lett..

[29] Fitzpatrick S, King A, Ryan S. Blockage of TLR4 or NLRP3 inhibits IL-1ß activation in response to intermittent hypoxia. Eur Respir J. 2020;56(suppl 64). https://erj.ersjournals.com/content/56/suppl_64/4734.

[30] Tsutsui H, Imamura M, Fujimoto J, Nakanishi K (2010). The TLR4/TRIF-mediated activation of NLRP3 inflammasome underlies endotoxin-induced liver injury in mice. Gastroenterol. Res. Pract..

[31] Yao X, Yan L, Yao L, Guan W, Zeng F, Cao F (2014). Acrylamide exposure impairs blood-cerebrospinal fluid barrier function. Neural. Regen. Res..

[32] Zorumski CF, Izumi Y (2012). NMDA receptors and metaplasticity: Mechanisms and possible roles in neuropsychiatric disorders. Neurosci. Biobehav. Rev..

[33] Tokuda K, Izumi Y, Zorumski CF (2013). Locally-generated acetaldehyde contributes to the effects of ethanol on neurosteroids and LTP in the hippocampus. Neurol. Clin. Neurosci..

[34] Alcocer-Gómez E, de Miguel M, Casas-Barquero N, Núñez-Vasco J, Sánchez-Alcazar JA, Fernández-Rodríguez A (2014). NLRP3 inflammasome is activated in mononuclear blood cells from patients with major depressive disorder. Brain Behav. Immun..

[35] Onat UI, Yildirim AD, Tufanli Ö, Çimen I, Kocatürk B, Veli Z (2019). Intercepting the lipid-induced integrated stress response reduces atherosclerosis. J. Am. Coll. Cardiol..

[36] Bo N, Yilin H, Haiyang Y, Yuan Y (2020). Acrylamide induced the activation of NLRP3 inflammasome via ROS-MAPKs pathways in Kupffer cells. Food Agric. Immunol..

[37] Leow-Dyke S, Allen C, Denes A, Nilsson O, Maysami S, Bowie AG (2012). Neuronal Toll-like receptor 4 signaling induces brain endothelial activation and neutrophil transmigration in vitro. J. Neuroinflammation.

[38] Coll RC, Robertson AAB, Chae JJ, Higgins SC, Muñoz-Planillo R, Inserra MC (2015). A small-molecule inhibitor of the NLRP3 inflammasome for the treatment of inflammatory diseases. Nat. Med..

[39] Gaidt MM, Ebert TS, Chauhan D, Schmidt T, Schmid-Burgk JL, Rapino F (2016). Human monocytes engage an alternative inflammasome pathway. Immunity.

[40] Groß CJ, Mishra R, Schneider KS, Médard G, Wettmarshausen J, Dittlein DC (2016). K+ efflux-independent NLRP3 inflammasome activation by small molecules targeting mitochondria. Immunity.

[41] Swanson KV, Deng M, Ting JPY (2019). The NLRP3 inflammasome: Molecular activation and regulation to therapeutics. Nat Rev. Immunol..

[42] Fan Y, Du L, Fu Q, Zhou Z, Zhang J, Li G (2018). Inhibiting the NLRP3 inflammasome with MCC950 ameliorates isoflurane-induced pyroptosis and cognitive impairment in aged mice. Front. Cell Neurosci..

[43] Zong C, Hasegawa R, Urushitani M, Zhang L, Nagashima D, Sakurai T (2019). Role of microglial activation and neuroinflammation in neurotoxicity of acrylamide in vivo and in vitro. Arch. Toxicol..

[44] Brydges SD, Broderick L, McGeough MD, Pena CA, Mueller JL, Hoffman HM (2013). Divergence of IL-1, IL-18, and cell death in NLRP3 inflammasomopathies. J Clin Invest..

[45] Hanamsagar R, Torres V, Kielian T (2011). Inflammasome activation and IL-1β/IL-18 processing are influenced by distinct pathways in microglia. J. Neurochem..

[46] Bellinger FP, Madamba S, Siggins GR (1993). Interleukin 1 beta inhibits synaptic strength and long-term potentiation in the rat CA1 hippocampus. Brain Res..

[47] Loscher CE, Mills KHG, Lynch MA (2003). Interleukin-1 receptor antagonist exerts agonist activity in the hippocampus independent of the interleukin-1 type I receptor. J. Neuroimmunol..

[48] Ross FM, Allan SM, Rothwell NJ, Verkhratsky A (2003). A dual role for interleukin-1 in LTP in mouse hippocampal slices. J. Neuroimmunol..

[49] Arend WP, Malyak M, Guthridge CJ, Gabay C (1998). Interleukin-1 receptor antagonist: Role in biology. Annu. Rev. Immunol..

[50] Schneider H, Pitossi F, Balschun D, Wagner A, del Rey A, Besedovsky HO (1998). A neuromodulatory role of interleukin-1beta in the hippocampus. Proc. Natl. Acad. Sci. USA.

